# Single center, retrospective evaluation of requiring 48 hours versus 24 hours before a pharmacist-driven protocol-based IV to PO conversion of azithromycin

**DOI:** 10.1017/ash.2025.8

**Published:** 2025-02-26

**Authors:** Abraham Felix, Winifred Pardo, Wilbert Fuerte, Laura Morales, Timothy P. Gauthier

**Affiliations:** 1 Department of Pharmacy, Homestead Hospital - Baptist Health South Florida, Homestead, FL, USA; 2 Department of Pharmacy, Baptist Health South Florida, Miami, FL, USA

## Abstract

Shortening a pharmacist-driven policy to allow a switch from IV to PO azithromycin after 24 hours instead of 48 hours led to 26% increase in oral azithromycin days of therapy (*P* < 0.001) and was associated with a shorter length of stay.

Antimicrobial stewardship represents a collaborative effort that involves the expertise of all disciplines to ensure optimal care for patients.^1^ In the daily functions of an antimicrobial stewardship program, pharmacists routinely play a key role in streamlining clinical care, which includes intravenous (IV) to oral (PO) interchanges that may be completed in collaboration with prescribers or per institutional policy without the need for prescriber approval.^2^ Infectious Disease Society of America (IDSA) guidelines on antimicrobial stewardship support IV to PO programs, and there is a healthy body of literature in support of this intervention type for improving clinical care.^3,4^ Conversion to oral therapy reduces the need for venous access, simplifies the medication administration process, can expedite hospital discharge, and is associated with reduced healthcare costs.^4^

There are innate inter-facility differences with regard to how IV to PO conversion programs are managed and which criteria must be met to allow for per-protocol conversion by pharmacists without contacting the prescriber. Within a single facility standardization (eg, no switch until day 3) and consistency (ie the same criteria for all eligible agents) may be a simpler approach for staff, but exceptions may be considered for certain antimicrobials. For example, azithromycin has adequate oral bioavailability for routine oral use and is not often used as definitive monotherapy for severe infections in hospitalized patients. In turn, consideration may be given to allow for greater flexibility in per-protocol IV to PO conversion with azithromycin.^5^ This is in contrast to an antibiotic such as ceftriaxone which often is used to treat severe infections and lacks a direct oral counterpart.

The purpose of this study was to explore the impact of shortening a pharmacist-driven per-policy requirement from 48-hours to 24-hours before allowing IV to PO conversion of azithromycin without contacting the prescriber.

## Methods

This was a retrospective, single-centered, quasi-experimental quality improvement project conducted in a 147-bed not-for-profit community hospital where dosing of azithromycin is most commonly 500 mg. In June 2023 the per-policy automatic change by pharmacist from IV to PO azithromycin was edited to allow for switch after 24 hours (one dose) of treatment rather than 48 hours. Different than the standard per-policy IV to PO criteria (eg, hemodynamic instability, positive blood culture, nonfunctioning GI tract), the expedited switch was not allowed for patients in a critical care unit but was allowed for patients with leukocytosis or a fever in the last 24 hours but was currently afebrile (see supplemental information). In effect, the pharmacist could change patients meeting criteria to oral azithromycin after the first IV dose was given in the emergency department, as the patient was transitioned to acute care. The primary endpoint of the project was the proportion of PO azithromycin days of therapy (DOT) adjusted to 1000 patient days out of all systemic azithromycin DOTs adjusted to 1000 patient days 5 months before and after the change. A minimum monthly threshold goal for the project overall was set to be 50% for the proportion of oral azithromycin DOTs. Data for this were extracted from Vigilanz electronic clinical decision support system which extracts data from Cerner Millennium. To secondarily examine the sustainability of the intervention the proportion of azithromycin oral DOTs were extended out 12 months.

A qualitative component to the study was undertaken to secondarily evaluate pre/post quality of care, for which adult (age ≥ 18 yr) patients who received at least one dose of IV or PO azithromycin between July-October 2023 were eligible. Exclusion criteria included pregnancy, incarceration, or not eligible for IV to PO switch within 5 days of azithromycin initiation. Patients in a critical care area not eligible for the expedited IV to PO switch were eligible for the qualitative analysis. Patients were identified from a Cerner Millennium Discern Analytics 2.0 report. Secondary endpoints included in the pre- and post-qualitative assessment included pharmacist intervention, median length of stay in days, changed back to IV after intervention of IV to PO, readmission within 30 days, and 30-day all cause in-hospital mortality. Per-protocol IV to PO conversion was included in the pharmacist intervention data.

For statistical analysis, categorical data were analyzed using chi-square, and descriptive statistics were used for demographics. For all calculations, a two-tailed P-value of < 0.05 was considered statistically significant. All collected data was analyzed using SAS 9.4 and/or R 4.1.2. This study was approved for exemption from the institutional review board.

## Results

**Figure 1.**
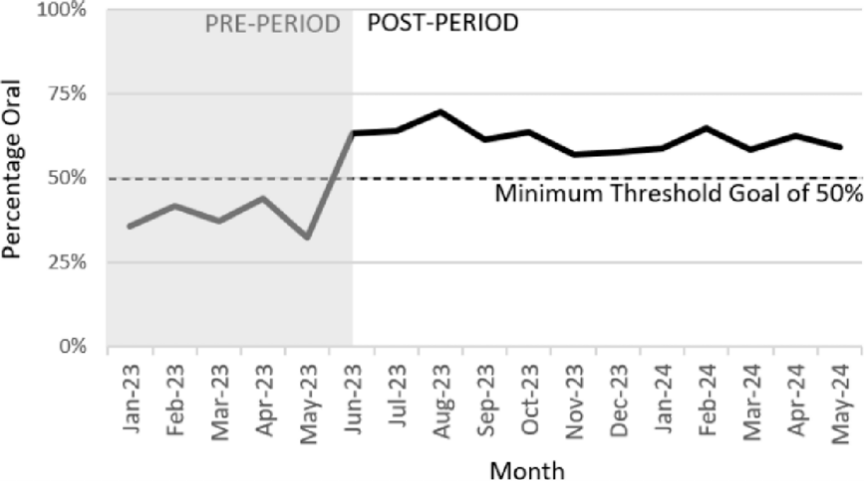
Percentage of oral azithromycin days of therapy before and after policy change.

In the 5-months leading up to the intervention, 38% of azithromycin DOT were for the oral product (506 PO of 1332 total DOT) versus in the 5-months after the intervention, 64% of azithromycin DOT were for the oral product (777 PO of 1213 total DOT), *P* < 0.0001. The percentage of monthly PO azithromycin DOT extending 12-months out after intervention implementation is displayed in figure 1.

For the qualitative analysis, 100 patients were included in each group. The median age was 69 years in both groups (*P* = 1). Critical care admission was present for 15% of patients in the pre group versus 16% of patients in the post group (*P* = 0.85). The most common indications were pneumonia (58% pre vs 65% post, *P* = 0.03) and chronic obstructive pulmonary disease (COPD) exacerbation (20% pre vs 25% post, *P* = 0.39). Pharmacist intervention was present for 52% of pre cases and 93% of post cases (*P* < 0.001). There were no patients in the pre group who were transitioned back to IV, while two patients were transitioned back to IV in the post group. Of these two patients, one was due to provider preference and the other was due to newly documented fever. Median length of stay was 4 days in the pre group versus 2.5 days in the post group (*P* = 0.002). Readmission at 30 days occurred in 17% of pre patients and 13% of post patients (*P* = 0.55). 30-day all-cause mortality occurred in 3% of patients within each arm (*P* = 1).

## Discussion

Utilization of oral azithromycin improved substantially following implementation of a more flexible per-policy IV to PO conversion by pharmacists and was not associated with any negative consequences in the qualitative assessment. For a facility with a substantial number of new IV azithromycin orders each week we feel the results represent a meaningful improvement, as demonstrated by exceeding the 50% minimum threshold goal in the post-period for the proportion of oral azithromycin monthly DOTs. These findings additionally support the critical role pharmacists play in fundamental stewardship activities when empowered by institutional policies, as demonstrated by the increase in pharmacist interventions.

The statistically significant reduced length of stay from 4 to 2.5 days in the post-period, was an important finding, other IV to PO conversion programs have been associated with reduced length of stay.^4^ However, given that azithromycin is often accompanying broad-spectrum beta-lactams, we did not anticipate more rapid transition to oral azithromycin would be associated with a shorter length of stay.

Although single-centered, it is likely that the indications azithromycin was prescribed for (mainly pneumonia and COPD exacerbation) align with the experience of other hospitals. That stated, the initiative may be best suited for facilities where a substantial volume of orders for IV azithromycin are received and where IV to PO is not yet fully optimized. Additionally, concern for the longevity may be a consideration, given this study period was just 5 months in the post-period. It is clear that continuous pharmacist engagement within the IV to PO conversions would be critical to the long-term success of a program such as this, where data are used to engage staff on an ongoing basis. Locally this is being supported by integration of clinical decision support technology with routine antimicrobial stewardship program monitoring.

In a time where resources for antimicrobial stewardship programs may be limited and antimicrobial resistance poses major threats to human health, a sustained commitment to fundamental antimicrobial stewardship interventions through practical protocol-based interventions such as this may support optimal patient outcomes.

## Supporting information

Felix et al. supplementary material Felix et al. supplementary material

## Supplementary material

For supplementary material accompanying this paper visit http://doi.org/10.1017/ash.2025.8. click here to view supplementary material
