# Supplementary material for: Single center, retrospective evaluation of requiring 48 hours versus 24 hours before a pharmacist-driven protocol-based IV to PO conversion of azithromycin
Source: Antimicrob Steward Healthc Epidemiol. 2025 Feb 26;5(1):e65. doi: 10.1017/ash.2025.8 (PMC11869072; doi:10.1017/ash.2025.8)
Supplement: Felix et al. supplementary material [file S2732494X25000087sup001.docx]

**Supplemental appendix**

Criteria for expedited and standard automatic intravenous to enteral azithromycin conversion by pharmacist.

**Expedited Criteria for Automatic Intravenous to Enteral Azithromycin Conversion By Pharmacist**

*Patients may be converted automatically by the pharmacist from intravenous to enteral azithromycin at the time of first ordering the intravenous formulation if the following are met. If the expedited criteria are not met, automatic conversion may only be done when standard criteria are met.*

INCLUSION ELIGIBILITY

□ Expedited automatic pharmacist conversion to enteral not allowed if patient is in or going to an Intensive Care Unit location

□ Expedited automatic pharmacist conversion to enteral allows for exceptions to the standard requirements, including:

- Expedited automatic pharmacist conversion to enteral allowed prior to reaching 24 hours of intravenous azithromycin coverage (note one dose equates to 24 hours of coverage)
- Expedited automatic pharmacist conversion to enteral allowed if patient has leukocytosis
- Expedited automatic pharmacist conversion to enteral allowed if patient is currently afebrile but has been febrile in the last 24 hours (conversion not allowed if currently febrile or recent use of acetaminophen)
- Expedited automatic pharmacist conversion to enteral allowed if patient has blood culture drawn and it is negative, but it has been less than 24 hours since specimen collection
- Expedited automatic pharmacist conversion to enteral not allowed if patient is hemodynamically unstable (not an exception, included for clarity)

EXPEDITED CONVERSOIN WORKFLOW

The process for patients meeting expedited criteria is as follows:

1. One dose of intravenous azithromycin profiled consistent with start date/time of prescribers intravenous order. If patient received a dose of intravenous already (e.g., in the Emergency Department), the patient will be directly converted to enteral.
2. An enteral order will be profiled for the remainder of the course, to start at the time of the next dose

*Patients not eligible for expedited conversion will be routinely assessed for potential intravenous to enteral conversion per the standard criteria protocol.*

**Standard Criteria for Automatic Intravenous to Enteral Antibiotic Conversion By Pharmacist**

INCLUSION CRITERIA

Patients who **meet all** of the following criteria are eligible for automatic pharmacist conversion from intravenous to enteral of azithromycin:

□ Intact/ functioning gastrointestinal tract

□ Able to tolerate oral or enteral feedings (e.g., oral diet, at least one liter of oral fluid daily)

□ Able to adequately absorb medications via the oral, gastric tube, or nasogastric tube route

□ Taking other medications orally

□ Patient has received at least 24 hours of intravenous antimicrobial therapy

□ Patient is afebrile without antipyretics for at least 24 hours

□ WBC is within normal range and/or trending towards normal range

□ No positive blood cultures (if blood cultures have been drawn)

□ Stable or improving radiographic findings (when applicable)

□ Hemodynamic stability

EXCLUSION CRITERIA

Patients **meeting any one** of the following criteria are not eligible for automatic pharmacist conversion from intravenous to enteral azithromycin.

*NOTE: Patients not meeting automatic pharmacist conversion criteria but who are reasonable candidates for the switch may be changed from intravenous to enteral route azithromycin only after discussion and agreement with the ordering provider.*

□ Physician ordered medication not to be switched to enteral route

□ Patient not taking anything by mouth (NPO status)

□ Malabsorption syndrome (e.g., obstruction, ileus, persistent nausea, vomiting or diarrhea in the last 24 hours, short bowel syndrome, motility disorder of the gastrointestinal system, gastroparesis)

□ Grade three or four mucositis (e.g., ulcers, extensive erythema – patients cannot swallow solid diet or alimentation is not possible)

□ Active gastrointestinal bleed

□ Patient at increased risk for aspiration (e.g., difficulty swallowing, decreased or loss of consciousness, seizures). Check swallow study if applicable.

□ Continuous nasogastric suction or nasogastric output above 150 mL for two or more times in a 24-hour period

□ Serious or life-threatening infection (e.g., meningitis, intracranial abscess, bacteremia, infective endocarditis, osteomyelitis, septic arthritis, orbital cellulitis)

□ Patient is septic, including any of the following: on vasopressors, heart rate above 90 beats per minute, respiratory rate above 20 breaths per minute, systolic blood pressure below 90 mmHG, documented sepsis or septic shock, other variables indicating criteria for sepsis (*e.g.*, activation of sepsis power plan)

□ Immunocompromised status (e.g., ANC below 1,000 cells/mm^3^, leukopenia with WBC below 4,000 cells/mm^3^)
